# Ocular Surface Changes in Prostaglandin Analogue-Treated Patients

**DOI:** 10.1155/2019/9798272

**Published:** 2019-12-10

**Authors:** Wencui Shen, Bingqing Huang, Jin Yang

**Affiliations:** ^1^Tianjin Eye Hospital & Eye Institute, Tianjin Key Laboratory of Ophthalmology and Visual Science, Nankai University, Tianjin, China; ^2^State Key Laboratory of Experimental Hematology, Institute of Hematology and Blood Disease Hospital, Chinese Academy of Medical Sciences and Peking Union Medical College, Tianjin, China

## Abstract

Glaucoma is the second leading cause of blindness globally. Reducing intraocular pressure (IOP) has been acknowledged to be the main therapy for glaucoma. Prostaglandin analogues (PGAs) have become the first-line therapy for patients with glaucoma due to their powerful efficacy for lowering (IOP). However, usage of PGAs can also cause several notable side effects, including the changes in ocular surface. The relationship between PGAs and ocular surface changes is complicated and still remains unclear. In the present review, we summarize the recent studies of the effects of PGAs on ocular changes as well as the possible mechanisms that might provide new considerations during clinical medication.

## 1. Introduction

Glaucoma is the second leading cause of blindness worldwide. It has been estimated that the number of patients with glaucoma will reach up to 79.6 million in 2020 [1]. Currently, it has been well recognized that intraocular pressure (IOP) is the key risk factor and the unique therapeutic target for glaucoma. The cause of IOP rise is due to the impairment of the outflow system [2]. Topical medication is mostly used for the initial treatment of glaucoma. The prostaglandin analogues (PGAs) have become widely used as the first-line strategy to reduce elevated IOP in glaucoma patients. With extensive clinical application of PGAs, it has been found that PGAs could influence ocular surface [3]. Except for some side effects (e.g., conjunctiva hyperemia, eyelash changes, and cystoid macular oedema), changes in corneal biomechanical properties, reduction of central corneal thickness (CCT), and ocular surface diseases (OSDs) related with preservatives of PGAs have also greatly drawn our attentions.

Among the side effects, some of the ocular surface changes have close correlation with IOP measurement and the progression of glaucoma, which highlights the clinical significance of themselves. The relationship between IOP and CCT has long been realized, and thinner CCT leads to the development of open-angle glaucoma (POAG) [4]. IOP and CCT are related to corneal biomechanical properties [5]. In addition, preservative, a relatively necessary element of PGAs, also makes a contribution to the changes in ocular surface, inducing complex inflammatory mechanisms and causing both allergy and toxicity [6].

In this review, we focused on the effect on the cornea and conjunctiva of PGA-treated individuals, with the following aspects summarized: corneal biomechanical properties, CCT, conjunctiva, wound healing, and dry eye. Interactions exist among these topics, encouraging further thinking and studies.

## 2. Corneal Biomechanical Properties

Recently, many studies have suggested that corneal biomechanical properties may prove to be important evidence of glaucoma progression, which could aggravate deformation of the optic nerve surface [7].

### 2.1. Corneal Hysteresis (CH)

CH is the resistance to corneal deformation, which is defined as the difference between the air-jet pressure at inward and outward applanation [8–10]. It has been confirmed that a low CH has a close relationship with advanced glaucoma damage and glaucoma progression [8]. More and more studies have demonstrated that topical PGAs could lead to alternation in CH of patients with glaucoma. A prospective case-control study of 108 eyes of POAG patients showed that, under PGA treatment, IOP values decreased and CH significantly increased, in which the parameters of each group were measured with an ocular response analyzer (ORA) before treatment and at 6-month intervals [11]. Indeed, earlier investigations have indicated that eyes with a higher IOP have a lower CH, and treatment of decreasing IOP may induce an opposite change in CH [12, 13]. With more attention to baseline IOP and CH, Agarwal and his colleagues indicated that although CH is lower in individuals with higher IOP, a lower CH could be an indicator of a powerful reduction of IOP under PGA treatment [14]. However, opposite points were found in patients under chronic PGA treatment during a prospective case-control study, which clarified that the cessation of chronic PGAs was associated with significant increase in CH, and that reinitiation of treatment could reverse the effect [15]. In addition, a 3-month animal research has recently been carried out with the result of no influence on CH of travoprost treatment. Therefore, the duration and cessation of PGA medication has also become an important factor in CH changes, and more rigorous studies are needed in this subject.

### 2.2. Corneal Resistance Factor (CFR)

CFR, another biomechanical property, reflects the overall resistance of the cornea to deformation, which may influence the measurement and the proper adjustment of IOP [12, 16]. Several studies have focused on the changes of CFR along with CH. Panagiotis Tsikripis and his colleagues made it clear that CFR did not show significant change in the PGA-treated group [11]. It is believed that CFR is correlated with CCT, regarding as an indicator in predicting glaucoma damage [17, 18]. However, PGAs were certificated to induce reversible reduction in CFR in those patients who accepted PGA monotherapy for at least 1 year [15]. The previous evidence improves our warning when assessing accuracy IOP of POAG patients under chronic PGA therapy, and we are in urgent need of more studies in animals and humans to deeply clear our points.

## 3. Central Corneal Thickness

Many studies have focused on the importance of central corneal thickness (CCT), a potential risk factor in glaucoma and ocular hypertension. CCT has been shown as the strongest predictive factor for visual field loss and optic nerve head changes of glaucoma [17]. However, corneal thickness affects Goldmann applanation tonometry; therefore, CCT and IOP both cannot be regarded as completely independent risk factors.

Several clinical studies reported that PGA therapy could decrease CCT [18–21]. Torsten Schlote and colleagues performed a consecutive, interventional case series including 74 patients/136 eyes with glaucoma, in which they found one-year treatment with travoprost led to a significant reduction of CCT [18]. Similar results were shown in three groups that accepted monotherapy with PGAs (latanoprost 0.005%, travoprost 0.004%, or bimatoprost 0.03%) for 6 months, and no significant difference was found among these three groups. Furthermore, Birt and colleagues indicated that the relationship between CCT and IOP lowering was negative at 12 weeks after initiating therapy, but a significant association was detected over a 24-week duration of medication [22]. Therefore, we could generally conclude that PGAs can increase CCT, but we could not make sure the precise follow-up period when the obvious effect of PGAs exhibited. Even so, CCT evaluation along with the IOP measurements at all visits can provide beneficial information for setting target IOP levels and defining “nonresponse” [23, 24].

Moreover, as we know, CH, CFR, and CCT are closely positively correlated [25]. CH, as above described, is a direct measurement of cornea biomechanics, whereas CCT, one of them, affects CH [11]. Roman Meda and colleagues confirmed that IOP underestimation was necessary in lower CH eyes [15]. CFR is also a useful predictor in glaucoma progression, which may be correlated with CCT [17, 18]. Further studies with a larger group and longer follow-up would be meaningful to confirm these opinions.

## 4. Conjunctival Modification

Conjunctival hyperemia, the most common side effect of PGAs, is regarded as the result from nitric oxide-mediated vasodilatation in the conjunctiva [26–28]. It is believed that the inflammation process is not involved in the reversible hyperemia. However, more and more patients preferred to pause the medication of PGAs [29, 30]. At present, several studies have focused on the toxicity of BAK (benzalkonium chloride), a mostly used preservative in eye drops. Although different kinds of PGAs contain different concentrations of BAK, it is recognized that conjunctival hyperemia is not a direct consequence of BAK toxicity. Latanoprost, with the highest concentration of BAK, causes a lower incidence of hyperemia [31].

Meanwhile, more and more in vitro and in vivo studies aim to clear the influence of PGAs on the conjunctiva. Eun Joo et al. revealed that BAK-induced cytotoxicity is dose-dependent in primary human conjunctival fibroblast cells [32]. Hong Liang and his colleagues indicated that ocular surface immunity played a necessary role in conjunctiva hyperemia, in which conjunctiva-associated lymphoid tissue (CALT) was involved [33–35]. They observed CALT reaction after BAK-containing eye drops treated by in vivo confocal microscopy (IVCM), characterized by inflammatory cell infiltration in the dome and intrafollicular layers and by cell circulation inside the lymph vessels [36]. A recent study about CALT in chronic OSD revealed that similar changes as increased lymphoid cells within the diffuse layer, follicles, and interfollicular spaces appeared in different stimulating environment [37]. Another interesting phenomenon observed by laser scanning confocal microscopy (LSCM) is that the mean area of epithelial microcysts increased in PGA-treated glaucomatous patients without medication history, which may predict the rising outflow of the transconjunctival aqueous humor [38]. Moreover, tear proteomics analysis showed increased levels of MMP-1, MMP-3, and MMP-9 and decreased levels of TIMP-1 and TIMP-2 in the PG-treated group [39]. Coincidently, MMP-1 and MMP-3 were upregulated in conjunctival biopsies of latanoprost-treated eyes, which may be related with reduced extracellular matrix deposition in the conjunctiva stroma [40]. It is generally recognized that the increased level of MMPs was involved in the process of ECM synthesis [41–44], which might have a better effect on the outcome of glaucoma filtering surgery [45].

## 5. Wound Healing

Above all else, clinically, a delay in corneal wound healing caused by PGAs seems to be another notable problem [46]. As we know, damage to the cornea can result in scarring, causes visual defects, and even leads to a complete loss of vision. Although components of PGAs, not only preservatives but also pH values, osmolarity, and so on, have latent toxicity [47], insufficient studies have been performed in this field, most of which still remain on the cellular level and animal experiment. Model of corneal erosion in rabbit was established by Pinheiro et al. [48], following which they measured the corneal healing process, and the corneal toxicity caused by the four PGAs was observed and evaluated. They concluded that monoprost could delay corneal healing, and BAC might be the main factor of corneal toxicity of Xalatan. Another similar research was done in rat debrided corneal epithelium, from which the investigators discovered that travoprost with sofZia seemed less toxic to the corneal epithelium [49]. Therefore, BAC could slow the corneal healing progress, due to its stimulatory effect on epithelial cell death [49–51] and its inducing effect in inflammation and apoptosis [52].

However, cationic oil-in-water emulsions (CE), a carrier of lipophilic drugs [53], has been found to improve the signs and symptoms of dry eye disease, which made researchers focus on the effectiveness and safety of preservative-free latanoprost 0.005% cationic oil-in-water emulsion (latanoprost-CE) [54]. Both in vitro and in vivo models showed the beneficial effect of CEs in corneal healing, whereas the adverse effect of BAC [55]. Thus, latanoprost-CE may have a longer-term prospect than BAC-based PGAs. More effective new preparations are needed, and clinical studies are also in need to ensure the advantages and safety of them.

## 6. Dry Eye Disease

Dry eye disease, one of the most common eye diseases [56], was redefined by the Asia Dry Eye Society as follows: “dry eye is a multifactorial disease characterized by unstable tear film causing a variety of symptoms and/of visual impairment, potentially accompanied by ocular surface damage.” As discordance between symptoms and signs of dry eye has been recognized [57], studies focused on the neurological or psychiatric factors in the development of dry eye have raised our concern [58, 59]. In addition, some researchers have proposed the concept of ocular surface microenvironment in both diagnosis and treatment of dry eye [60, 61].

More and more studies have demonstrated that a large proportion of patients who accepted the topical medication of PGAs suffer from ocular surface disease (OSD) [62–64]. And half of them complained that they suffered the discomfort of dry eye, which could be attributable to the active components as well as to the preservatives with the mechanisms of allergy, toxicity, and inflammation [65, 66]. However, some researchers have compared dry eye between glaucoma patients receiving PGA monotherapy and nonglaucoma subjects, and their results showed no significant difference between the two groups, suggesting that adverse ocular effects induced by PGA might be minimal [67]. And in a cross-sectional study, Teresa Rolle et al. indicated that tafluprost had no effect on tear stability comparing with preservative-free timolol, and the active component itself of both the groups may lead to ocular surface impairment [63]. Another prospective study showed that beta blockers induced more serious impact on the ocular surface mainly due to the active substances [68]. So, it seems that PGAs have better tolerance than beta blockers. Indeed, a similar conclusion was given in pediatric patients with glaucoma or ocular hypertension [69].

On the contrary, meibomian gland dysfunction (MGD) is generally an important cause of dry eye [70]. And several new techniques to evaluate the morphology and function of meibomian glands have been developed [71]. Long-term topical medication of PGAs can cause obstructive type of MGD [72]. Interestingly, Luca et al. have observed the morphological changes in meibomian glands induced by antiglaucoma medication through LSCM. They showed more significant modifications in the preserved PGA group than the preservative-free PGA group [73]. More studies should be carried out between PGAs and meibomian gland.

In addition, LSCM as the most reliable method for analyzing goblet cell (GC) in vivo can indentify GC morphology in glaucoma-related OSD [74]. GC loss may influence the ocular surface immune tolerance that is observed in dry eye [75]. A recent study showed the detrimental effect on GCs caused by antiglaucoma medications, including PGAs [76]. Moreover, preoperative dendritic cell (DC) density and goblet cell density are evaluation parameters correlated to the filtration surgery outcome in glaucomatous patients [77]. So, it is necessary to consider the GC and DC conditions of patients during glaucomatous medication and surgery.

Besides the effective components, the preservatives of PGAs can also cause dry eye. Among them, most of the studies have focused on the relationship between PGAs with different concentrations of BAK and dry eye. BAK, which is usually used at a concentration of 0.01% (range, 0.005–0.02%) in topical multidose solutions, has effective antimicrobial activity and high affinity for membrane proteins [78]. It is proved that BAK could hasten the drying of the tear film and aggravate preexisting dry eye [79, 80]. An open-label multicentre study of 158 patients has shown that preservative-free tafluprost was better tolerated than preserved latanoprost, in which tear break-up time (TBUT), Schirmer's test, and fluorescein staining were used to evaluate the severity of ocular surface discomfort [81]. Another similar study performed by Giménez-Gómez et al. indicated that switching from preservative PGAs to preservative-free tafluprost could relieve the symptoms of dry eye [82]. Therefore, preservative-carrying prostaglandin aggravates ocular dryness symptoms, and patients with ocular surface discomforts may benefit from switching to a preservative-free prostaglandin. However, Jess T. Whitson and his colleagues found that there were no significant differences in TBUT and corneal staining among PGAs with different concentration of BAK, including bimatoprost (0.005% BAK), latanoprost (0.02% BAK), and travoprost (sofZia) [47]. Certainly, long-term investigations are needed to evaluate the ocular surface tolerability and optimize the preservative systems in glaucoma medications.

Furthermore, other different kinds of antiglaucomatous agents also contribute to the development of dry eye [62, 83–85], mainly induced by preservatives [62, 68]. And it is reported that dry eye risk may be increased by more than two types of glaucoma medications, particularly in female patients in Taiwan population [86]. Thus, larger and further clinical researches are needed to evade the territorial limitation.

## 7. Conclusion

In summary, there is indeed a close relationship between PGAs and ocular surface. Despite the effectiveness of controlling IOP, PGAs have a considerable influence on ocular surface of those subjects, whether in anatomical structure of cornea, or in the pathological change in OSD. We have concluded existing studies and opinions in the above five parts. Herein, we try our best to do a warning that IOP measurement should be adjusted due to the corneal properties during the clinical therapy. And a series of ocular surface disorders caused by PGAs should be recognized during follow-up treatment. Therefore, these disadvantages of PGAs encourage us to invent more precise methods recording medicated IOP and to develop more effective and safer antiglaucoma drugs.
